# Dietary Intake by Dutch 1- to 3-Year-Old Children at Childcare and at Home

**DOI:** 10.3390/nu6010304

**Published:** 2014-01-08

**Authors:** Jessica S. Gubbels, Lieke G. M. Raaijmakers, Sanne M. P. L. Gerards, Stef P. J. Kremers

**Affiliations:** Department of Health Promotion, NUTRIM School for Nutrition, Toxicology and Metabolism, Maastricht University Medical Centre, PO Box 616, Maastricht 6200 MD, The Netherlands; E-Mails: jessica.gubbels@maastrichtuniversity.nl (J.S.G.); sanne.gerards@maastrichtuniversity.nl (S.M.P.L.G.); s.kremers@maastrichtuniversity.nl (S.P.J.K.)

**Keywords:** childcare, day-care, dietary intake, dietary journal, nutrition, observation, overweight, parent, toddler

## Abstract

The goal of the current study was to assess dietary intake in a large sample (*N* = 1016) of Dutch toddlers (1–3 years old), both at childcare and at home. Dietary intake during two weekdays was recorded using an observation format applied by childcare staff for intake at childcare, and partially pre-coded dietary journals filled out by parents for intake at home. Children’s intake of energy, macronutrients and energy balance-related food groups (fruit, vegetables, sweet snacks, savoury snacks) were compared with Dutch dietary guidelines. In addition, differences between the dietary intake by various subgroups (based on gender, age, childcare attendance, socio-economic status of childcare centre) were explored using multilevel regression analyses, adjusting for nesting of children within centres. Energy intake was high relative to dietary guidelines, and children consumed more or less equal amounts of energy at home and at childcare. Dietary fibre, fruit and vegetable and snack intakes were low. Intake at childcare mainly consisted of carbohydrates, while intake at home contained more proteins and fat. The findings imply various opportunities for childcare centres to improve children’s dietary intake, such as providing fruit and vegetables at snacking moments. In addition, the findings underline the importance of assessing dietary intake over a whole day, both at childcare and at home, to allow intake to be compared with dietary guidelines.

## 1. Introduction

Worldwide, at least 42 million children under the age of five were overweight in 2010, and these numbers are expected to continue to increase [1]. Childhood overweight is a major risk factor for several chronic conditions such as cardiovascular diseases and type 2 diabetes mellitus [2]. Moreover, childhood overweight is known to track into adulthood, in that overweight children often remain overweight or obese during later life [3]. Dietary intake plays a crucial role in the development of overweight [4]. Dietary habits are often established at a young age [5] and maintained throughout life [6,7,8], indicating the urgency of increasing our understanding of the origin and development of dietary habits in young children.

In Europe, over half of the toddlers (below primary school age) attend some form of childcare or educational facilities [9]. It has been recommended that a child in full-time childcare (*i.e.*, 8 h or more per day) should consume one half to two-thirds of his or her daily dietary intake at childcare [10], indicating the importance of childcare for the development of children’s dietary habits. Childcare use has been found to be associated with an increased overweight risk throughout childhood (e.g., [11,12,13]). Furthermore, various studies have shown that children attending childcare often do not meet dietary intake recommendations: they may consume excess energy [14] and excessive amounts of total fat [14,15], saturated fat [15,16] and sweets [14]. In addition, they are not consuming sufficient amounts of fruit [16], vegetables [14,16,17] and dietary fibre [18]. However, several of these studies were limited to dietary intake at childcare [16,19,20], ignoring the intake at home. As such, they have to rely on the estimated proportion of the dietary intake that takes place at childcare. Since dietary intake at home is not known, these studies assume the composition of the meals to be stable throughout the day and do not take into account possible compensation behaviour at home. The studies that have taken account of both dietary intake at home and intake at childcare [14,15,17,18,21,22,23] mostly had small sample sizes (*N* < 200) [14,17,18,21,22], assessed dietary intake at childcare through the parents [18], or only examined specific meals instead of dietary intake during a whole day [18]. In addition, the majority of the studies examining dietary intake have been conducted in the United States [14,15,16,17,18,20,21], with only a few from Europe [19,22,23].

The current study aimed to assess dietary intake in terms of energy, macronutrients and the food groups of fruit, vegetables, sweet snacks and savoury snacks, both at childcare and at home, in a large sample (*N* = 1016) of Dutch toddlers (aged 1–3 years). In addition, it explored the dietary intake in various subgroups (according to gender, age, childcare attendance and socio-economic status (SES) of the childcare centre’s neighbourhood).

## 2. Methods

### 2.1. Respondents and Procedure

Ethical approval for this study was not required according to Dutch law, since the current research did not involve invasive procedures, and thus did not fall under the Dutch Medical Research Involving Humans Act (Wet Medisch-Wetenschappelijk Onderzoek met Mensen) [24]. All childcare centres in the Netherlands were approached to participate in the study from March 2011 onwards. Several strategies were used to recruit childcare centres. A direct mailing of letters was sent to addresses acquired by purchasing commercially available addresses. In addition, a digital mailing was sent, and childcare centers were recruited at conferences and through appointments at the head offices of the childcare organizations to which the centres belonged. If the head office was interested, the recruitment was continued at the individual centres. All childcare centres were allowed to participate. Sometimes a centre decided not to participate citing reasons such as that it would be too much effort, the centre had been closed down, the parent committee did not agree or management had changed. The 112 childcare centres that responded before August 2013 were included in the study. Data collection started as soon as a childcare centre consented to participate. All parents of the children aged 1 to 3 years from these centres were invited to participate. A total of 2788 children participated. All parents of participating children provided informed consent. Children’s dietary intake was recorded on two entire weekdays, randomly chosen during one week, both at home using food diaries and at childcare using observations.

### 2.2. Assessment of Dietary Intake

In the Netherlands, children attending childcare usually consume their breakfast at home. Subsequently, they consume a morning snack, lunch and afternoon snack at childcare, and their dinner again at home.

#### 2.2.1. Dietary Intake at Childcare

Staff at the childcare centre was instructed by a dietician to record the dietary intake of each of the participating children on a poster. The poster was a partially pre-coded dietary record, providing a list of the most common products that might consumed at each different eating moment. For instance, it showed a list of sweet snacks, beverages and fruits commonly consumed at snacking moments in the Netherlands. In addition, it provided space at each eating moment to record any other products consumed which were not on the standard list. There was a separate column on the poster for each participating child, where their intake could be recorded. Childcare staff was asked to specify the type of product (e.g., whether the milk product consumed was milk, chocolate milk, butter milk or yoghurt drink), the unit (e.g., whether it was a cup or a bottle), and the amount (*i.e.*, number of units).

The first eating moment (the snacking moment of the first observation day) was recorded together with the dietician, at which point the childcare staff received detailed instructions from the dietician on how to record the dietary intake. If the childcare staff were still uncertain about any aspects, these would also be explained by the dietician. During the rest of that day, and on the second observation day, the childcare staff filled in the poster for all eating moments at the centre (*i.e.*, morning snack, lunch and afternoon snack).

An additional questionnaire was filled in by the childcare staff together with the dietician to record further information regarding the meals and foods offered at the centre, such as the standard portion size used for certain products (e.g., how many mL were in the cups used) and the type and brand of particular products (e.g., whether regular or low-fat margarine was used and what brand).

#### 2.2.2. Dietary Intake at Home

Parents were also asked to record their child’s dietary intake at different eating moments at home during the two measurement days (*i.e.*, breakfast, dinner including dessert, and anything consumed after dinner, including anything consumed during the night). The questionnaire consisted of a partially pre-coded food journal, providing a list of common products that might be consumed at each different eating moment. For instance, the food journal listed the bread and bread products, butters or margarines, sandwich toppings, fruit, porridges and beverages that are often consumed at breakfast in the Netherlands. In addition there was space to record any other products consumed at each eating moment that were not on the standard list. Parents were asked to specify the type of product (e.g., whether bread was white or brown), the unit (e.g., whether it was a slice of bread or a roll), the brand, and the amount (*i.e.*, number of units).

### 2.3. Assessment of Background Characteristics

Children’s age (rounded off to whole months), gender and the number of days they attended childcare were asked for in the parental questionnaire. The socio-economic status (SES) score of the population catered for by each childcare centre was derived from the centre’s postal code. These SES scores are standardized scores per neighbourhood, reflecting educational level, income, and work status of the residents of that neighbourhood [25]. The SES scores were recoded into low, medium and high, based on tertile cuts of all scores in the Netherlands [25].

### 2.4. Processing of Dietary Intake Data

Only respondents for whom complete dietary intake data were available (for both measurement days, both at childcare and at home) were retained in the analyses. Of the 2788 children participating in the entire study, 1016 (43.7%) provided complete dietary intake data for both measurement days, both at home and at childcare. Of the 1773 children without complete data, the majority (75.0%) had complete data at childcare, but data at home was only available for 1 day or no days at all. Furthermore, 24.0% had only attended childcare on the day, so data for two complete days at childcare could not be provided, and 1.0% only provided data for intake at home, but not for childcare. The 1016 children with complete data were included in the final analyses.

The observed and reported dietary intake data of the children were entered by the dieticians in the FoodFigures Program [26] separately for each of the six eating moments (breakfast, morning snack, lunch, afternoon snack, dinner, evening snack). The amounts consumed as reported by the childcare staff and parents were recalculated by this program into weights and volumes using the procedures on measures and weights of the Dutch nutrient database [27] where necessary (e.g., using a standardized weight for a slice of bread). Amounts of half or a quarter of a portion were also recalculated by the program. As the focus of the current paper was on dietary intake, the average intake per day of the following nutrients was calculated by the program, based on the Dutch nutrient database [27]: energy (in kcal), proteins (in energy percent (en%)), carbohydrates (en%), total fat (en%), saturated fat (en%), unsaturated fat (en%) and fibre (grams (g)). In addition, intake from the following energy balance-related food groups was calculated: fruit (g), vegetables (g), sweet snacks (g; including sweets (e.g., jelly candy, liquorice, marshmallows), chocolate, cookies (e.g., butter cookies) and pastry (e.g., cake, pie)) and savoury snacks (g; including salty snacks (e.g., potato chips) and fried snacks (e.g., fried meats)).

### 2.5. Data Analyses

All analyses were conducted using SPSS 20.0 [28]. *p*-Values < 0.05 were considered statistically significant. Independent *t*-tests and chi-square tests were conducted to compare the background characteristics (children’s age, gender, childcare attendance, and the childcare centre’s SES) of the children in the final sample with those of children who had incomplete data and were thus excluded.

Descriptive statistics were used to explore the children’s background characteristics and total dietary intake. In addition, children’s total dietary intake was compared with the dietary guidelines for toddlers from the Netherlands Nutrition Centre (Voedingscentrum; see Table 1) [29]. The overall dietary guidelines applied in the current study were specific Dutch guidelines [29]. The dietary guidelines for toddlers from the Netherlands Nutrition Centre refer to the guidelines for a healthy food choice of the Netherlands Nutrition Centre for a balanced dietary intake for children of one year and older. These guidelines are therefore used as a benchmark source for nutrient and food group analyses. Next, children’s dietary intakes at home and at childcare were analysed separately, as well as their intakes at different eating moments (breakfast, morning snack, lunch, afternoon snack, dinner and evening snack).

**Table 1:** Total dietary intake by toddlers, compared to national guidelines (*N* = 1016).

Dietary Intake	Actual Total Dietary Intake Mean (SD)	Dietary Guideline ^a^	Percentage of Children Meeting the Guideline
Energy (kcal)	1285.1 (238.2)	1200	37.5% ^b^
Carbohydrates (en%)	55.7 (5.2)	≥45	98.1%
Proteins (en%)	14.3 (2.1)	≤20	99.2%
Fat			
*Total (en%)*	30.0 (4.8)	25–40	83.2% ^c^
*Saturated (en%)*	10.7 (1.8)	≤15	98.6%
*Unsaturated (en%)*	16.6 (3.7)	-	-
Dietary fibre (g)	12.5 (2.7)	≥15	17.2%
Fruit (g)	124.1 (61.9)	≥150	27.5%
Vegetables (g)	64.7 (36.5)	≥50–100	69.3% ^d^ 17.0% ^e^
Sweet snacks ^f^ (g)	13.5 (12.0)	-	-
Savoury snacks ^g^ (g)	0.7 (4.0)	-	-

Notes: en% = energy percent, g = grams, mL = millilitres, *N* = number of children, SD = standard deviation; ^a^ Nutritional guidelines from the Netherlands Nutrition Centre ([29]). No specific guidelines are available for unsaturated fats and snacks; ^b^ 10% deviation from the guideline allowed (*i.e.*, 1080–1320 kcal). Below the guideline: 18.8%; above the guideline: 43.7%; ^c^ below the guideline: 14.9%; above the guideline: 2.0%; ^d^ *N* (%) meeting the guideline of 50 g/day; ^e^ *N* (%) meeting the guideline of 100 g/day; ^f^ Including sweets, chocolate, cookies and pastry; ^g^ Including salty snacks and fried snacks.

In addition, the intakes by subgroups of children based on gender (boys *vs*. girls), age (2, 3 or 4 years old), childcare attendance (up to 2 days *vs*. 3 or more days a week) and the childcare centre’s SES (low, medium or high) were explored. Multi-level linear regression analyses were conducted to examine the associations between these background variables and the dietary intake variables, corrected for the nesting of children within childcare centres and the background variables.

## 3. Results

Of the 1016 children, 54.8% (*N* = 554) were male. The average age was 2 years and 1 month (SD = 10 months), with 313 1-year-olds (34.1%), 330 2-year-olds (36.0%) and 274 3-year-olds (29.9%). On average, the children went to childcare for 2.4 days per week (SD = 0.6). A total of 24.5% of the children attended childcare centres that were located in low-SES neighbourhoods, 28.2% in medium-SES neighbourhoods, and 47.3% in high-SES neighbourhoods.

Children included in the final sample attended childcare for slightly more days a week than children with incomplete dietary intake data (2.4 *vs*. 2.1, *p* < 0.001). There were no other significant differences in background characteristics (age, gender, childcare SES) between children who did or did not drop out.

### 3.1. Dietary Intake and Guideline Compliance

Table 1 lists the total dietary intake of the toddlers in the current study, as well as the number and percentage of children complying with the dietary guidelines of the Netherlands Nutrition Centre [29]. About one third of the children (37.5%) complied with the guidelines regarding energy intake. A smaller group (18.8%) consumed less than the recommended amount of energy, while most children (43.7%) consumed more energy than recommended.

The vast majority of the children met the guidelines with regard to macronutrients (carbohydrates, proteins and fat). However, only 17.2% of the children consumed sufficient dietary fibre. This was also reflected in the low percentages of children consuming sufficient fruit and vegetables. Snack intake (both sweet and savoury) was generally low.

### 3.2. Dietary Intake at Childcare and at Home

Children consumed more or less equal amounts of energy at home and at childcare. However, while their intake at childcare mainly consisted of carbohydrates, a relatively larger proportion of the intake at home consisted of proteins and fat (see Table 2). Furthermore, children consumed most of their fruit at childcare, and most of their vegetables at home. Sweet snacks were mostly eaten at childcare.

### 3.3. Dietary Intake at Different Eating Moments

Table 3 shows the children’s dietary intake at different eating moments, as well as the intake as a proportion of total daily intake (from different meals). The percentage of children consuming food at each of the eating moments was very high (98.6%–99.8%), except for evening snacks, which were consumed by only 62.8% of the children.

The main energy sources were lunch and dinner, together accounting for over half (57.4%) of the total energy intake. The snacking moments were very high in carbohydrates, while the main meals contained relatively larger proportions of proteins and fat. The main sources of dietary fibre were the main meals. Most fruit was consumed during the morning snacking moment, while the afternoon snacking moment often involved sweet snacks (e.g., cookies, sweets, pastry). The evening snacking moment involved a relatively large proportion of saturated fat compared to the other snacking moments.

**Table 2:** Dietary intake by toddlers at childcare and at home (*N* = 1016).

Dietary Intake	Dietary Intake Mean (SD)	
	At Childcare	At Home
Energy (kcal)	652.2 (169.9)	633.5 (171.9)
Carbohydrates (en%)	62.4 (6.6)	49.1 (8.4)
Proteins (en%)	12.0 (2.4)	16.8 (3.7)
Fat		
*Total (en%)*	25.6 (5.6)	34.1 (7.8)
*Saturated (en%)*	9.6 (2.3)	11.7 (2.8)
*Unsaturated (en%)*	13.4 (3.9)	19.5 (6.1)
Dietary fibre (g)	6.6 (1.8)	5.9 (2.1)
Fruit (g)	98.7 (46.0)	25.5 (41.4)
Vegetables (g)	10.9 (21.6)	53.8 (33.4)
Sweet snacks ^a^ (g)	10.3 (9.4)	3.3 (7.9)
Savoury snacks ^b^ (g)	0.2 (1.5)	0.5 (3.7)

Notes: en% = energy percent, g = grams, mL = millilitres; ^a^ Including sweets, chocolate, cookies and pastry; ^b^ Including salty snacks and fried snacks.

### 3.4. Dietary Intake in Subgroups

Overall, there were few differences in total dietary intake between boys and girls. Boys consumed significantly more energy than girls (1304.8 *vs*. 1264.6 kcal, *p* < 0.01), as well as more dietary fibre (12.7 *vs*. 12.2 g, *p* < 0.02). There were no significant differences between boys and girls in intake specifically at childcare.

Intake of energy was significantly higher among older children, both specifically at childcare and for the day as a whole (see Table 4). Dietary fibre intake also increased with age, mainly at childcare. Sweet snacks intake increased with age, although at childcare, this increase was only significant between 2 and 3 years, while the increase in overall sweet snacks intake was only significant between 1 and 2 years. Total savoury snack intake increased between the ages of 1 and 2 years.

Children who attended childcare for 3 or more days a week had a higher total vegetables consumption (73.3 *vs*. 62.0 g, *p* < 0.02), and consumed more savoury snacks (1.1 *vs*. 0.6 g, *p* < 0.04; results not tabulated) than children attending childcare for 2 days or less. Childcare attendance was not significantly related to specific dietary intake at childcare.

**Table 3:** Dietary intake by toddlers at different eating moments.

Dietary Intake	Dietary Intake Mean (SD)											
	Breakfast at Home (*N* = 1006)		Morning Snack at Childcare (*N* = 1010)		Lunch at Childcare (*N* = 1014)		Afternoon Snack at Childcare (*N* = 1002)		Dinner at Home (*N* = 1013)		Evening Snack at Home (*N* = 638)	
	Mean (SD)	% of total intake	Mean (SD)	% of Total Intake	Mean (SD)	% of Total Intake	Mean (SD)	% of Total Intake	Mean (SD)	% of Total Intake	Mean (SD)	% of Total Intake
Energy (kcal)	237.7 (87.1)	18.1	126.1 (51.8)	9.6	378.5 (122.9)	28.8	147.6 (67.9)	11.2	358.8 (120.0)	27.2	66.8 (63.5)	5.1
Carbohydrates (en%)	54.9 (10.6)	14.5	85.4 (10.8)	22.6	49.6 (7.7)	13.2	81.2 (12.8)	21.5	43.0 (11.6)	11.4	63.6 (23.1)	16.8
Proteins (en%)	15.0 (4.6)	19.6	5.2 (3.5)	6.8	16.2 (3.9)	21.3	6.4 (4.6)	8.4	19.1 (5.4)	25.1	14.3 (12.4)	18.8
Fat												
*Total (en%)*	30.0 (10.0)	20.5	9.5 (8.5)	6.5	34.5 (7.1)	23.5	12.5 (10.2)	8.5	37.9 (11.1)	25.9	22.1 (16.1)	15.1
*Saturated (en%)*	11.5 (4.2)	20.8	3.1 (4.6)	5.6	12.9 (3.1)	23.3	5.0 (5.4)	9.1	12.1 (3.6)	21.9	10.7 (8.9)	19.3
*Unsaturated (en%)*	15.7 (7.8)	20.8	4.1 (4.5)	5.4	18.3 (5.2)	24.2	5.5 (6.2)	7.3	22.7 (9.0)	30.0	9.3 (11.1)	12.3
Dietary fibre (g)	2.6 (1.2)	20.5	1.5 (0.9)	11.8	3.9 (1.4)	30.7	1.2 (0.9)	9.5	3.0 (1.3)	23.6	0.5 (0.9)	3.9
Fruit (g)	7.5 (22.6)	5.9	70.5 (51.7)	55.9	1.2 (8.6)	1.0	30.7 (45.0)	24.3	12.8 (27.6)	10.2	3.4 (14.2)	2.7
Vegetables (g)	0.3 (3.0)	0.5	0.1 (1.0)	0.2	5.1 (15.8)	7.8	2.8 (10.2)	4.3	56.4 (32.5)	87.0	0.1 (2.0)	0.2
Sweet snacks ^a^ (g)	1.2 (4.3)	8.7	3.6 (6.4)	26.1	0.2 (1.3)	1.4	7.3 (8.2)	52.9	0.8 (4.1)	5.8	0.7 (3.0)	5.1
Savoury snacks ^b^ (g)	0.0 (0.5)	0.0	0.0 (0.5)	0.0	0.0 (0.0)	0.0	0.3 (1.6)	42.9	0.4 (3.7)	57.1	0.0 (0.4)	0.0

Notes: en% = energy percent, g = grams, mL = millilitres; ^a^ Including sweets, chocolate, cookies and pastry; ^b^ Including salty snacks and fried snacks.

**Table 4:** Dietary intake differences based on age.

Dietary Intake	Dietary Intake at Childcare				Dietary Intake during a Whole Day			
	1-Year-Olds Mean (SD) *N* = 313	2-year-olds ^a^ Mean (SD) *N* = 330	3-Year-Olds Mean (SD) *N* = 274	Significance ^b^	1-Year-Olds Mean (SD) *N* = 313	2-Year-Olds ^a^ Mean (SD) *N* = 330	3-Year-Olds Mean (SD) *N* = 274	Significance ^b^
Energy (kcal)	570.7 (150.1)	662.3 (158.2)	726.8 (170.9)	***	1165.9 (209.1)	1305.9 (210.1)	1400.4 (231.7)	***
Carbohydrates (en%)	62.5 (7.1)	63.0(5.8)	62.3 (6.3)		55.7 (5.5)	56.0 (5.1)	55.8 (4.8)	
Proteins (en%)	12.1 (2.8)	11.8 (2.1)	12.0 (2.3)		14.5 (2.4)	14.1 (1.9)	14.2 (2.1)	
Fat								
*Total (en%)*	25.4 (5.8)	25.2 (5.2)	25.6 (5.3)		29.8 (5.1)	29.9 (4.7)	30.0 (4.4)	
*Saturated (en%)*	9.6 (2.4)	9.4 (2.1)	9.5 (2.1)		10.7 (1.9)	10.6 (1.7)	10.6 (1.6)	
*Unsaturated (en%)*	13.3 (4.0)	13.3 (3.8)	13.5 (3.7)		16.5 (3.9)	16.5 (3.5)	16.7 (3.5)	
Dietary fibre (g)	6.1 (1.7)	6.6 (1.9)	7.2 (1.8)	***	12.3 (2.6)	12.3 (2.6)	13.0 (2.7)	* ^c^
Fruit (g)	99.8 (45.8)	98.4 (49.5)	98.9 (45.0)		122.6 (62.3)	124.3 (63.4)	127.8 (63.1)	
Vegetables (g)	13.5 (23.7)	9.8 (20.5)	9.8 (20.7)		68.5 (36.3)	61.9 (36.1)	64.6 (38.2)	
Sweet snacks ^d^ (g)	8.8 (8.3)	9.9 (8.6)	12.1 (11.4)	* ^e^	11.3 (10.6)	13.6 (12.2)	16.0 (13.5)	* ^c^
Savoury snacks ^f^ (g)	0.1 (1.3)	0.2 (1.5)	0.5 (1.9)		0.3 (1.9)	1.1 (5.8)	0.9 (3.5)	* ^c^

Notes: en% = energy percent, g = grams, mL = millilitres; ^a^ Reference category; ^b^ Adjusted significance from multivariate multi-level regression analyses, adjusted for gender, childcare attendance and socioeconomic status score of the childcare centre; ^c^ Only significant for the 1-year-olds; ^d^ Including sweets, chocolate, cookies and pastry; ^e^ Only significant for the 3-year-olds; * *p* < 0.05, ** *p* < 0.01, *** *p* < 0.001; ^f^ Including salty snacks and fried snacks.

There were no differences in overall intake between childcare centres with different SES. With regard to the specific intake at childcare, children at high-SES childcare centres consumed significantly less fruit (93.0 g) than children at medium- and low-SES centres (106.2 g and 101.2 g, respectively, *p* < 0.04). On the other hand, they consumed significantly more vegetables at childcare (14.9 g) compared to children from medium- and low-SES centres (7.2 g and 7.3 g, respectively, *p* < 0.01). Children at low-SES childcare centres consumed significantly lower amounts of energy (619.0 kcal) than those at medium- and high-SES centres (679.8 and 652.9 kcal, respectively, *p* < 0.04). Finally, children at low-SES centres consumed significantly less savoury snacks (0.4 g compared to 0.1 g and 0.3 g in medium- and high-SES centres, respectively, *p* < 0.05; results not tabulated).

## 4. Discussion

The current study assessed dietary intake at childcare and at home in a large sample (*N* = 1016) of Dutch toddlers (1–3 years) who attended childcare. Energy intake was high relative to dietary guidelines, while dietary fibre, fruit and vegetable intakes were low. Snack intake (both sweet and savoury) was low. In 2005 and 2006, a national food consumption survey was conducted among toddlers in the Netherlands including children who attended childcare as well as those who did not. The dietary intake among the 2- to 3-year-olds (*N* = 788) from that survey was very similar to the intake we found in the current sample, specifically as regards the intake of energy, all macronutrients, dietary fibre and fruit (differences all <5%) [30]. This indicates that the overall dietary intake by children attending childcare does not seem to be very different from that by children not using childcare. Compared to the national survey, however, children in the current sample appeared to consume far less snacks (13.5 g of sweet snacks *vs*. 47 g in the national survey; and <1 g of savoury snacks *vs*. 3 g in the national survey) and more vegetables (64.7 g compared to 40 g in the national survey) [30].

It is unclear why there were such considerable differences with regard to vegetable and snack intakes, but not with regard to any other dietary intake measures. Perhaps the fact that the current study included 1-year-olds can partly explain these differences, especially with regard to snacks, because the 1-year-olds in the current sample consumed significantly less snacks than the older children. Furthermore, it should be noted that the current study did not include days on which the children did not attend childcare. It is possible that the children from the current sample had different intake patterns during a full day at home. However, a previous study by Ziegler and colleagues [18], which compared lunch and snacking moments at childcare with the corresponding eating moments during a full day at home, found only slight differences in intake between these two locations, which were only significant for the afternoon snacking moment: at home, children seemed to consume a bit more protein and fat in the afternoon. Furthermore, Ziegler *et al*. [18] reported more frequent consumption of salty snacks in the afternoon at home than at childcare. A study by Lehtisalo [23] that compared the dietary intake of children cared for at childcare with that of children cared for at home found lower vegetable consumption and higher sweet pastry consumption by the children cared for at home. These findings are in line with the deviating vegetable and snack consumption in the current sample compared to the national survey [30]. A final explanation for the differences between the current study and the national survey may regard the fact that the current study did not include weekend days. Several studies have shown that children’s dietary intake is generally less healthy on weekend days (e.g., [22,23,31]), possibly explaining the lower snack consumption and higher vegetable intake in the current study.

In line with previous research among young children (e.g., [30,32]), the children in the current sample skipped very few meals and snacking moments: 98.6%–99.8% of the children consumed food at each of the eating moments (except for an evening snack, consumed by 62.4%). As regards the quality of children’s diets, the macronutrient content of their diets seemed to be very good, with 83.2% to 99.2% of the children meeting the guidelines for carbohydrates, proteins, total fat and saturated fat. Studies from the US found excess consumption of total and saturated fat in childcare [14,15,16], perhaps reflecting a cultural difference between the US and the Netherlands. However, in line with previous US studies [14,16,17,18], many children in the current sample did not consume sufficient dietary fibre, vegetables and fruit. Furthermore, almost half of the children consumed excess amounts of energy (*i.e.*, >1320 kcal), which is in line with previous research [14]. About equal amounts of energy were consumed at home and at childcare. Energy intake at the different eating moments in the current study was comparable to that found in US studies [18,20].

There were few differences in dietary intake between subgroups, both at childcare and in total. In line with previous research [19], boys consumed more energy than girls. In addition, boys consumed more dietary fibre. Concerns about children’s diet seemed to change with age: while younger children were more likely to consume insufficient dietary fibre, the older children often consumed more snacks and energy. These differences were visible specifically at childcare as well as during a whole day, with the exception of savoury snack intake, whose increase was only significant as regards intake during a whole day. With regard to childcare attendance, children who attended three or more days a week consumed more vegetables and savoury snacks, though not at childcare, indicating that this increased vegetable and snack intake took place at home. Furthermore, we found older children to consume more energy, dietary fibre, sweets and snacks. Despite the fact that The Netherlands Nutrition Centre recommends the same intake for children aged 1–4 in their guidelines [29], our results show that children within this age group have different needs. Children in the age of 1 may for example still be nursed which influences their dietary intake.

Although the overall consumption of snacks seemed to be low (13.5 g of sweet snacks and less than one gram of savoury snacks per day on average), the majority of the sweet snacks were consumed at childcare, especially during the afternoon snacking moment. Fruit was consumed especially during the morning snack at childcare, and to a lesser extent in the afternoon. This indicates an opportunity for childcare centres to improve children’s fruit consumption (which was too low for almost three quarters of the children), and at the same time even further lower snack consumption, by replacing the afternoon sweet snacks with fruit. Fruit consumption seemed to be especially low in high-SES childcare centres, while intake at home was not significantly different between childcare centres with different SES. Previous studies have repeatedly shown that children from low-SES families often consume less fruit (e.g., [33,34]). However, children from high-SES childcare centres in the current study also consumed more vegetables. It seems that high-SES childcare centres place more emphasis on vegetable intake, and less on fruit intake.

Various previous studies examining dietary intake at childcare have used the guidelines of the American Dietetic Association (that a child who spends a full day at childcare [*i.e.*, 8 h or more] should consume one half to two-thirds of his or her daily dietary intake at childcare [10]) to convert daily dietary intake guidelines into estimated guidelines for intake at childcare (e.g., [16,17,19]). However, such conversion into childcare-specific guidelines ignores the fact that the composition of meals and other eating moments is not stable throughout a day (e.g., the composition of a typical lunch is different from the composition of a typical dinner), as the current study shows. It therefore makes no sense to apply the same guidelines at home and at childcare. This underlines the importance of studies assessing dietary intake during a total day, both at home and at childcare, enabling comparison with daily intake guidelines.

The current study had several limitations. There was a relatively high percentage of incomplete cases (56.3%), although the final sample included in the study can still be considered very large (over 1000 children from over 100 different childcare centres) compared to previous studies. This large sample size also provided sufficient statistical power to correct the analyses for the multi-level structure of the data. Nonetheless, there was some selective drop-out with regard to longer childcare attendance. In addition, the data collection took place over a relatively long period (30 months), which could have influenced the results. A strength of the current study was that dietary intake was assessed both at childcare and at home, making it possible to compare the intake with dietary guidelines without having to estimate the proportion of intake taking place at childcare. However, dietary intake at childcare was observed and recorded by childcare staff, while dietary intake at home was self-reported by parents, possibly introducing bias. Moreover, the dietary intake assessment methodologies used in both settings (childcare and home) were not validated, and weekend days were not assessed in the current study. With regards the software used to recalculate intake, participants were not asked whether children consumed their entire plate/glass which may have biased the reported intake. However, the ability of the program to register amounts of half or a quarter of a portion helped accurate assessment of children’s intake. Moreover, the dietary intake in the current sample was very similar to the intake in a previous national survey, indicating that the assessment methods in the current study were probably sufficiently reliable.

## 5. Conclusions

In terms of energy balance, the main concern of children’s dietary intake in our study was their low fibre, vegetable and fruit consumption, and their high energy intake, putting them at risk for developing overweight. Childcare has a large potential to contribute to resolving these issues, for instance by offering fruit as a snack twice instead of once a day, and by providing vegetables during lunch and snacking moments. This could potentially also further lower snack consumption and thereby lower energy intake, thus also reducing the overweight risk. Previous studies have shown that childcare staff can have an important positive influence on children’s dietary intake at childcare, for example by serving sufficient healthy foods [16,35], preferably using a family serving style (in which the child can take healthy foods him/herself and can decide how much to take) [19,36,37] or indulgent feeding style (giving and offering seconds for healthy foods) [37,38]; by being a positive role model and eating healthy foods together with the children [19]; and by talking about healthy foods with the children [19].

As regards research, the current findings underline the importance of future research assessing dietary intake during a total day, both at home and at childcare. This enables comparison with daily guidelines, instead of having to convert these guidelines to improvised childcare-specific guidelines. In addition, intake should be assessed during a full day at home as well, including weekend days, to be able to compare intake at home and at childcare and to check for variability in children’s diets across locations.
